# Royal Medical and Chirurgical Society

**Published:** 1897-12-18

**Authors:** 


### ROYAL MEDICAL AND CHIRURGICAL SOCIETY.

### The Prevention of Enteric Fever.

We cannot at present refer to more than two of the points raised at the adjourned discussion on the prevention of typhoid fever held on Tuesday. Dr. Payne spoke very strongly in favour of dealing with the infection at the fountain head, and urged that, if the ideas at present current in regard to the etiology of the disease were correct, if, that is, every case originated directly or indirectly from a preceding case, to prevent the communication of the infection, in other words, to intercept the infection as it left the patient, would be enough to stamp out the disease. The problem, according to this view of the case, was to destroy the bacillus while leaving the body, and while it was within our reach, and above all, not to allow it to infect water or soil where it might grow, and whence it might spread; and the prevention of typhoid fever involves the protection of our water supplies from *all* forms of pollution by excreta, for to stop its growth then, when it had got such a foothold, seemed to be an almost hopeless task. Those, then, in charge of typhoid cases occupied a most responsible position, for on their intelligent efforts to ensure the disinfection of the discharges depended the stamping out of the disease. In this case prevention, like charity, began at home.

We need hardly point out that the importance of Dr. Payne's remarks lay in the fact that they were the proper and perfectly logical deduction from certain widely accepted premisses. Kill every microbe as it leaves its burrow and the breed will die. Unfortunately, even clinical experience tells us that many cases of typhoid fever wander about during a considerable portion of the time of their greatest infectivity, and as the application of modern tests tends to show that a certain proportion of the cases commonly returned as diarrhœa are really cases of typhoid fever, it becomes more and more obvious that the destruction of the excreta, even of every recognised case, must fail to "stamp out" the disease from our midst.

In regard to this aspect of the question the remarks made by Professor Kanthack were of great interest, for he showed that, looking at the matter from the laboratory standpoint, and taking the presence of the typhoid bacilli as being equivalent to the presence of infection, it is by no means true that the infectiveness of a typhoid case is limited to the period during which the fever manifests itself. It was generally recognised, he said, that the bacilli were found in the fæces and the urine, and also in the abscesses which sometimes occurred during the progress of typhoid fever. But it had been found that this by no means expressed the whole case, for the bacilli might be discovered in abscesses months, and even years, after attacks of typhoid fever, and also might persist for very long periods in the gall-bladder, whence they might gain access to the intestines, and presumably might give rise to further infection.

Thus it was made clear, so far as the bacteriologist may express an opinion (and if not he, who else can?) that typhoid patients may remain infectious long after recovery, and that apparently healthy people may be an entirely unsuspected source of contamination to soil and water.

The bearing of all this on the prevention of the disease is obvious, for it is clear that this is not to be accomplished by merely looking after the excreta of the sick. Truly, prevention, like charity, should begin at home; but, also like charity, it should not end there.

